# Restricted grouper reproductive migrations support community-based management

**DOI:** 10.1098/rsos.150694

**Published:** 2016-03-09

**Authors:** Peter A. Waldie, Glenn R. Almany, Tane H. Sinclair-Taylor, Richard J. Hamilton, Tapas Potuku, Mark A. Priest, Kevin L. Rhodes, Jan Robinson, Joshua E. Cinner, Michael L. Berumen

**Affiliations:** 1 Australian Research Council Centre of Excellence for Coral Reef Studies, James Cook University, Townsville, Queensland 4811, Australia; 2 CRIOBE—USR 3278, CNRS-EPHE-UPVD and Laboratoire d’Excellence ‘CORAIL’, 58 Avenue Paul Alduy, Perpignan Cedex 66860, France; 3 Red Sea Research Center, Division of Biological and Environmental Science and Engineering, King Abdullah University of Science and Technology, Thuwal 23955, Kingdom of Saudi Arabia; 4 Indo-Pacific Division, The Nature Conservancy, South Brisbane, Australia; 5 Kavieng Field Office, The Nature Conservancy, Kavieng, Papua New Guinea; 6 Marine Spatial Ecology Laboratory and Australian Research Council Centre of Excellence for Coral Reef Studies, School of Biological Sciences, University of Queensland, St Lucia, Queensland 4072, Australia; 7 MarAlliance, PO Box 283, San Pedro, Ambergris Caye, Belize

**Keywords:** fish spawning aggregation, Epinephelidae, movement ecology, marine protected areas, acoustic telemetry, marine reserve

## Abstract

Conservation commonly requires trade-offs between social and ecological goals. For tropical small-scale fisheries, spatial scales of socially appropriate management are generally small—the median no-take locally managed marine area (LMMA) area throughout the Pacific is less than 1 km^2^. This is of particular concern for large coral reef fishes, such as many species of grouper, which migrate to aggregations to spawn. Current data suggest that the catchment areas (i.e. total area from which individuals are drawn) of such aggregations are at spatial scales that preclude effective community-based management with no-take LMMAs. We used acoustic telemetry and tag-returns to examine reproductive migrations and catchment areas of the grouper *Epinephelus fuscoguttatus* at a spawning aggregation in Papua New Guinea. Protection of the resultant catchment area of approximately 16 km^2^ using a no-take LMMA is socially untenable here and throughout much of the Pacific region. However, we found that spawning migrations were skewed towards shorter distances. Consequently, expanding the current 0.2 km^2^ no-take LMMA to 1–2 km^2^ would protect approximately 30–50% of the spawning population throughout the non-spawning season. Contrasting with current knowledge, our results demonstrate that species with moderate reproductive migrations can be managed at scales congruous with spatially restricted management tools.

## 1. Introduction

Trade-offs between social and ecological goals are ubiquitous in human-populated ecosystems. For tropical small-scale fisheries management, understanding and evaluating these trade-offs is vital. Many coastal communities in developing countries are characterized by low incomes and high reliance on natural resources for livelihoods and food security [1]. Their needs, immediate and on-going, are directly reliant on the sustainable use of surrounding ecosystems, but this cannot come at excessive short-term social costs. In recent decades, community-based co-management programmes have proliferated throughout the tropics in an attempt to balance the needs of local stakeholders with fisheries management and conservation goals [2–4]. These programmes use existing community governance structures to establish and enforce fisheries management rules, and rely heavily on the support and compliance of local stakeholders. However, community-based fisheries management generally requires significant compromise, between scales of management which are ecologically precautionary and biologically meaningful [5], and those which are socially realistic [6,7].

For coral reefs, the most widely used community-based fisheries management tools are locally managed marine areas (LMMAs), whereby coastal communities limit or prohibit extractive or destructive practices within a defined area [4,8,9]. Although LMMAs are somewhat analogous with contemporary marine protected areas, they generally operate on more limited spatial and temporal scales [10], potentially reducing their effectiveness for conservation outcomes [7,11–13]. No-take LMMAs, where extractive and destructive practices are prohibited, are often particularly small; the median size of no-take LMMAs in the Pacific is less than 1 km^2^, and no-take LMMAs greater than 10 km^2^ are extremely uncommon [4]. Managing large, mobile coral reef fishes with such spatially limited tools may be problematic. However, evaluating LMMA effectiveness for such fishes has proved difficult, due in part to our limited understanding of their spatial ecology [14]. In one notable exception, however, a no-take LMMA prohibited fishing of a multi-species grouper (*Epinephelidae*) spawning aggregation, resulting in increased fish abundances at the site during the first 5 years of protection [15].

Many large fishes, including many grouper species, form transient fish spawning aggregations (FSAs) where otherwise relatively solitary and sedentary individuals migrate over varying distances to aggregation sites, and amass in markedly increased densities relative to non-reproductive periods [16]. The temporal and spatial predictability of FSAs combined with typically large increases in catchability make them attractive fishing opportunities. Extensive fishing of aggregations has led to the systematic collapse of FSAs globally [16,17]. Emerging evidence that FSAs contribute substantially to the larval supply of local populations [18] suggests that such FSA failures may result in localized population extinctions. The ecological importance of FSAs, combined with their limited and predicable spatial and temporal extent, make them attractive candidates for a wide range of management action, including community-based management. Conversely, the intrinsic vulnerability of many aggregating species and the potential for long-range migrations to and from FSAs [14,19] suggest that small no-take LMMAs which protect the aggregation site alone may be inadequate for many aggregating species. This is particularly true in scenarios where there is sustained fishing pressure outside of the FSA site, as the spawning population is vulnerable throughout the non-spawning season [20].

Here we focus on the brown-marbled grouper *Epinephelus fuscoguttatus* (Forsskål, 1775), a coral reef fish that forms FSAs throughout its range and is listed as Near Threatened on the IUCN Red List [21,22]. Analysis from the Great Barrier Reef, Australia, reveals life-history characteristics of *E. fuscoguttatus* which are associated with increased vulnerability to overfishing [23]; large size (growing to approx. 1 m in length), late-maturity (age at 50% female maturity—9 years), and longevity (maximum age 42 years) [24]. *Epinephelus fuscoguttatus* is a highly prized target of subsistence, artisanal and large-scale commercial fisheries throughout the Indo-Pacific, including Papua New Guinea [21,25–30]. Yet, information on the migratory movement of *E. fuscoguttatus* is limited to a single study where the total catchment area (i.e. total area from which all individuals are drawn) of an FSA was estimated at 100–175 km^2^ [19]. Although protection of the entire catchment of an FSA may be ecologically ideal, a community-based LMMA of this size would be socially untenable. More detailed information on migratory movement is required to effectively evaluate trade-offs between social and ecological conservation goals, and to inform management decision-making [14].

In this study, we combined acoustic telemetry and tag-returns with social surveys to assess the current effectiveness, and to suggest potential changes to increase the effectiveness, of a small LMMA in Papua New Guinea, established to protect a multi-species grouper FSA in 2004 [15].

Specifically, we sought to answer the following research questions:

(i) how much protection does the current no-take LMMA provide to the *E. fuscoguttatus* that have aggregated to spawn?
(ii) does the current LMMA provide protection to the *E. fuscoguttatus* population outside of their spawning periods? and
(iii) how do local stakeholders perceive the spatial expansion of the current no-take LMMA to include non-spawning areas, relative to other management options?

## 2. Material and methods

### 2.1 Social context and study site

**Figure 1.**
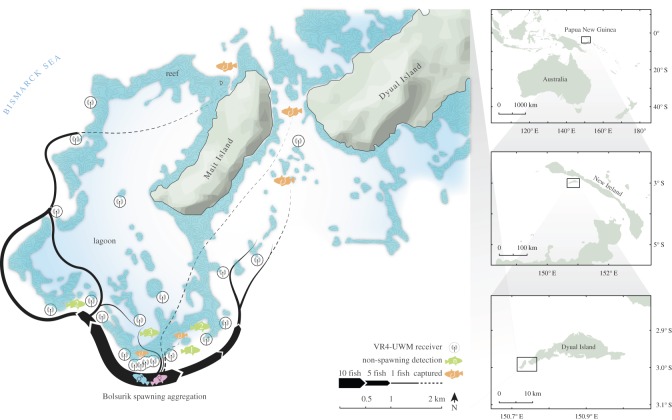
Map of Dyual Island, Papua New Guinea, showing *E. fuscoguttatus* migrations to and from the Bolsurik FSA site. Black lines represent movement of fish; either between acoustic receivers (solid), or between acoustic detection and the point of capture by the local fishery (dashed). Thickness of lines denotes the number of fish moving between points. Fish symbols represent locations of either detections during the non-spawning season (green; numbers denote number of fish detected), or capture by the local fishery (orange; all symbols represent a single fish).

Our study was conducted on the reefs surrounding Dyual Island, New Ireland Province, Papua New Guinea (figure 1). Here, as in many nations throughout the Asia-Pacific region, a customary tenure system devolves management rights to clan groups [4]. On Dyual Island, the customary tenure system is strong, centuries-old, and enshrined in national law, and thus represents the appropriate governance structure for managing natural resources [11,31,32]. A LMMA was established here in 2004 through a partnership between The Nature Conservancy and the local community, to protect a multi-species FSA located along a seaward facing reef promontory. Five years of protection of the FSA, locally known as Bolsurik, led to significant spawning population recoveries for two species of grouper (*Epinephelus polyphekadion* and *E. fuscoguttatus*) that aggregate here [15]. *Epinephelus fuscoguttatus*aggregate at the FSA site to spawn (as validated by significantly increased densities and the presence of gravid females) for approximately one week leading up to the new moon for four to five months annually, between March and July, with peak densities generally observed in May [15].

### 2.2 Acoustic range testing

Stationary acoustic range tests were conducted, prior to the deployment of the receiver array, using one Vemco (AMIRIX Systems Inc., Halifax, Canada) VR4-UMW receiver temporarily deployed seaward from the reef wall proximal to the FSA. Four fixed delay tags (V13-1x-A69-1601, 69 kHz, delay 60 s, Vemco) were activated at 15 s intervals to avoid signal collisions, attached to dive weights with sub-surface marker buoys for relocation, and deployed along the reef wall at distances of 50, 100, 150 and 200 m from the receiver. Over a period of 24 h, almost all transmissions were successfully detected at 50 m and 100 m from the receiver (94% and 86%, respectively), however, detection success dropped away sharply at greater distances (150 m, 46% success; 200 m, 3% success). This is consistent with comprehensive range testing of this tag type in reef environments which yielded a mean detection range (distance at 50% probability detection across times and habitat types) of 120 m [33]. Mobile range testing was conducted following deployment of the receiver array (see receiver array deployment, below). A diver carried a fixed delay tag (V13-1x-A69-1601, 69 kHz, delay 7 s, Vemco) during three surveys of the FSA site. The array provided good coverage of the site, with 96% of transmissions successfully detected on at least one receiver, and 64% detected on multiple receivers.

### 2.3 Receiver array deployment

Twenty VR4-UMW receivers were suspended from sub-surface marker buoys, at approximately 10 m depth, with antennae facing downwards, and moored to the reef substrate using stainless steel cables. Four receivers were deployed along the reef wall at the FSA site, with significant overlap in working range (45–54 m apart) to ensure complete coverage of the site (figure 1 (circles around receivers indicate working detection range)). Additionally, 11 receivers were deployed along the reef wall fringing the Mait lagoon, two receivers were moored proximal to patch reefs in the passage between Mait and Dyual islands, and three receivers were deployed proximal to patch reefs within the Mait lagoon (figure 1).

### 2.4 Tagging

Twenty-nine *E. fuscoguttatus* were captured at the FSA site, using baited hook-and-line, during the last quarter lunar phase of two months (May and June) of the 2013 spawning season. Individuals were weighed (to the nearest 0.1 g) and measured (total length (TL) to the nearest millimetre). If necessary, swim-bladders were deflated by inserting a sterile hypodermic needle posterior to the pectoral fin. Individuals were sexed by stripping (i.e. applying pressure to the abdomen) or by inserting a sterile cannula into the gonophore, to extract eggs or milt. Individuals were then fitted with passive acoustic random delay-coded tags (V13-1x-A69-1601, delay 100–140 s, 1120-day battery life, Vemco), inserted into the gut cavity through a small incision, which was then closed with non-absorbable sutures and treated with a topical antibiotic. Fish were administered with 50 mg kg^−1^ fish weight of a saline solution of the broad-spectrum antibiotic oxytetracycline, directly into the muscle tissue of the caudal peduncle, and then returned to the reef, approximately 20 m from the aggregation site. Transmitter activity was tested upon release using a VR100 acoustic receiver and VH165 omnidirectional hydrophone (Vemco).

Data were downloaded after a period of 2 years. Data for visitation to the FSA site were collated, with arrivals and departures expressed in days relative to the new moon. Following arrival at the FSA site, males were detected consistently (using the blanking threshold of 12 h) until departure and subsequent migration from the FSA site. Two females each went undetected for periods longer than 12 h during two visitations. During these periods, these fish either (i) went undetected despite remaining at the FSA or (ii) departed and returned to the site. Our data are insufficient to conclusively differentiate between these hypotheses. However, these fish were not detected on receivers proximal to the FSA site during these periods, and conversely were detected on proximal receivers during all intra-monthly migrations to and from the FSA site. This suggests that they did not vacate the FSA site during these periods, and these periods were thus not treated as departures from the site. Residence was calculated as the difference between arrival and departure in days. To facilitate comparison among years, aggregation month was expressed as the number of new moons since the preceding southern summer solstice (henceforth lunar month). Data from the tagging month for each individual were excluded from all analyses, to account for the potential short-term behavioural effects of the tagging procedure. All data passed inspections for deviations from homoscedasticity and normality prior to analyses.

### 2.5 Stakeholder surveys

Thirty-two household surveys were conducted in *tok pisin* (the *lingua franca*) on the western half of Dyual Island. Every second household (defined as a group living and eating together) was surveyed at the primary settlement in the study area (*n*=15), and at the six smaller coastal settlements (*n*=12, 1–4 surveys per settlement). Household members were approached in selected dwellings, and an interview was requested with either the male or female head of the household. Where they were unavailable, the household was missed and returned to at a later date. Five surveys were also opportunistically conducted with heads of households situated in the interior of the island, when heads of these households were available for interview within coastal settlements. It is likely that such households were under-represented in the sampling design; however, information from key informants suggested that members of households situated in the interior of the island used marine resources only sporadically, and as such were not major targets of this study. Survey respondents were asked whether they supported the Bolsurik LMMA in 2004 and 2014, using a Likert scale (1–5; actively oppose, passively oppose, neutral, passively support, actively support). They were then asked how the LMMA had affected their livelihood, their community and the environment, using a Likert scale (1–5; very negative, slightly negative, no effect, slightly positive, very positive). Respondents were then asked to suggest any changes that they felt would improve the current management. Finally, respondents were asked whether they had heard of, witnessed, or participated in poaching activities within the LMMA, whether they recognized the poachers and whether poachers suffered any consequences.

### 2.6 Statistical analyses

Arrival, departure and residence data at the FSA were each fitted with linear mixed-effects models (LMEMs) fit by maximum likelihood, with sex and lunar month as fixed factors, and year and individual as random factors. *p*-values were obtained by likelihood ratio tests of the full model against the model without the effect in question.

The complete FSA catchment area was estimated by fitting the minimum convex polygon which contained all recaptures and acoustic detections, using ArcGIS [34]. Maximum recorded migration distance (the greatest Euclidean distance from the FSA site to a point of acoustic detection or recapture) was calculated for each individual, using ArcGIS [34]. To investigate the potential effects of uneven sampling effort, correction factors of 1.5 and 2.0 were individually applied to a subset of these data, such that data from detections or recaptures during the non-spawning season (44%) remained unchanged, while data from acoustic detection during migratory periods (56%) were multiplied by the correction factor. This yielded three permutations of the dataset: (i) ‘uncorrected’, (ii) ‘1.5 corrected’, and (iii) ‘2.0 corrected’. Migration kernels were then separately fitted to these datasets (*n*=25). We proposed three candidate migration kernels to explain these data: the (i) Weibull, (ii) gamma, and (iii) lognormal kernels. Parameter sets for each distribution were estimated by maximizing the likelihood function:

$$L(\theta )=\prod_{i=1}^{n}f(d_{i}|\theta ),$$

where *d*_*i*_ are the *n* observations of migration distance and *θ* represents the parameter set of the function. An Akaike information criterion weighting analysis was performed on the resulting maximum-likelihood functions, fit to the migration data. The lognormal function provided the best fit of the uncorrected data, with an Akaike weight of 0.67, compared with the gamma and Weibull kernels, with Akaike weights of 0.21 and 0.11, respectively. The lognormal function also provided the best fit of the 1.5 corrected data, with an Akaike weight of 0.44, compared with the gamma and Weibull kernels, with Akaike weights of 0.31 and 0.25, respectively. The lognormal, gamma and Weibull functions provided relatively equivalent fits of the 2.0 corrected data, with Akaike weights of 0.33, 0.35 and 0.33, respectively. A 95% confidence interval was then calculated for each maximum-likelihood lognormal function using non-parametric bootstrapping, with 1000 bootsamples (figure 4).

Pearson product-moment correlation coefficients were computed to assess the relationships between migration distance and number of visits to the FSA, and between migration distance and total residence time at the FSA, over the duration of the study.

All analyses were conducted using the R software package [35]; LMEMs were fitted using the ‘lme4’ package [36]; functions were fitted to the migration distance distributions using the ‘fitdistrplus’ package [37]. The data and script files needed to execute all above analyses are publicly available (see ‘Data accessibility’ section).

## 3. Results

Twenty male (578–811 mm TL) and nine female (505–654 mm TL) *E. fuscoguttatus* were fitted with acoustic transmitters. Four males were removed from all analyses, owing to detection patterns suggestive of post-tagging mortality (lack of detections greater than 1 h post-tagging) or predation (uncharacteristic repeated, rapid movements along the reef for up to one week immediately post-tagging, with no further detections). Five acoustically tagged male *E. fuscoguttatus* were captured by local fishers as part of their regular fishing activity (i.e. not part of the study), but these tags were returned and the locations of the captures disclosed. One fish was poached from within the LMMA 55 days after tagging; the other fish were captured at distances of 0.6, 5.0, 5.4 and 6.0 km from the FSA 442, 623, 116 and 84 days after tagging, respectively.

### 3.1 Visitation to the fish spawning aggregation

**Figure 2.**
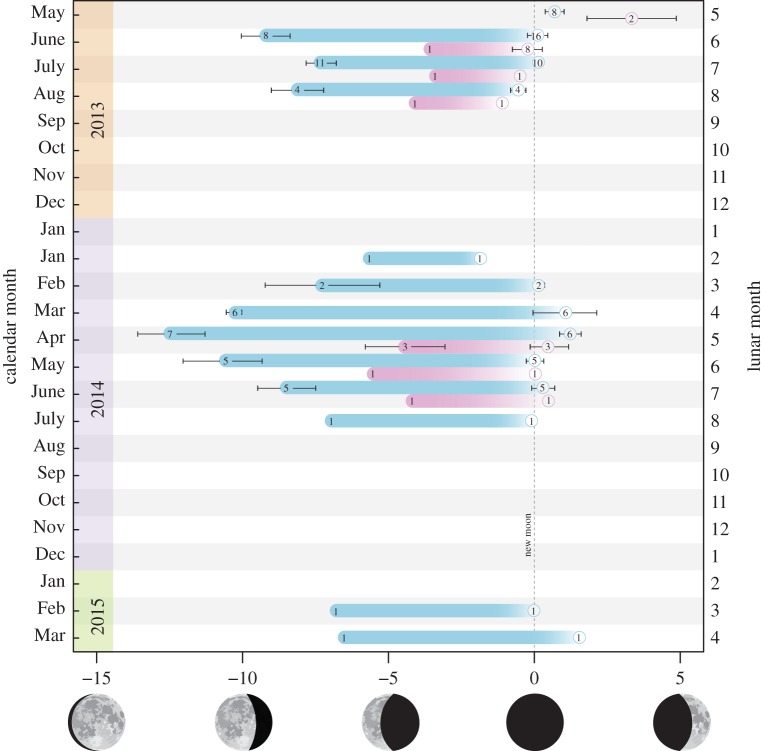
Visitation to the Bolsurik FSA site by acoustically tagged male (blue) and female (magenta) *E. fuscoguttatus*. Days (*x*-axis) are expressed relative to the new moon (vertical dashed line). Spawning month is expressed as calendar month (left *y*-axis), and as lunar month (number of lunar cycles following the previous southern summer solstice) (right *y*-axis). Mean arrival (filled circles), departure (open circles) and residence (filled bar) are displayed for each month. Numbers within circles represent number of tagged individuals present, and error bars represent standard errors.

Acoustic tagging data revealed that some tagged individuals were present at the FSA site during 13 lunar months of the 24 month study period. Although the number and length of visitations varied substantially between individuals (discussed in detail below), no tagged individuals remained at the FSA site either between spawning months, or between spawning seasons (figure 2). Over the 24 month study period, males spent a mean 35.66 (±7.48 s.e.; range, 6.30–88.66) days at the FSA site (and thus within the LMMA), compared with 11.3 (±1.11; range, 9.27–13.10) days for females.

Males returned to the FSA more frequently than females over the study period, (*t*_23_=2.89, *p*=0.008); less than half (44%) of the tagged females were redetected after their tagging month, compared with over three-quarters (78%) of the males. Of those redetected after their tagging month, males were detected at the FSA for a mean 2.64 (±0.27) lunar months during 2013 and 2.70 (±0.72) months during 2014, and females were detected at the FSA for a mean 1.75 (±0.48) lunar months during 2013 and 1.25 (±0.63) months during 2014. Males visited the FSA for up to six months in one spawning season. One male visited the FSA during 11 lunar months over the 24 month study period. Although data were more limited for females, two females visited the FSA for three consecutive months during a single season.

Males arrived at the FSA a mean 5.48 (±1.18) days before females ($\chi_{1}^{2}=21.77$, *p*<0.001), and resided a mean 5.76 (±1.57) days longer ($\chi_{1}^{2}=13.51$, *p*=0.001); departures were not significantly affected by sex ($\chi_{1}^{2}=0.32$, *p*=0.571; figure 2). Mean residence time at the FSA steadily increased from 5.84 (±1.79) days during the first month of the spawning season, to peak at 12.56 (±1.68) days mid-way through the season, before declining again to 7.53 (±1.77) days by the last month of the season ($\chi_{6}^{2}=96.98$, *p*<0.001; figure 2). Inter-monthly variation in residence was driven by fish arriving earlier ($\chi_{6}^{2}=66.29$, *p*<0.001), and departing later ($\chi_{6}^{2}=55.97$, *p*<0.001) mid-way through the season (figure 2). Residence, and arrival and departure time all varied substantially among individuals (explaining 75.4, 60.7 and 63.1% of model variances, respectively), although not among years (explaining 2.7, 10.1 and 0.0% of model variances, respectively).

### 3.2 Reproductive migrations

All tagged individuals were detected moving away from the FSA site (and thus out of the LMMA) between each spawning month, and between spawning seasons. Individuals dispersed from the FSA to the west (44%), east (48%) or directly over the fringing reef and into the lagoon (8%) along common migratory corridors (figure 1). For individuals that made multiple migrations to and from the aggregation site (16 migrations by three females; 64 migrations by 13 males), detected migration routes were rarely identical. Two alternative (although not mutually exclusive) hypotheses could explain this: (i) individuals use differing routes between spawning months, or (ii) tagged individuals show high fidelity to migration routes but pass some receivers undetected. The data support the latter, as illustrated by the following example. One male was detected on four receivers during migrations across six spawning months. During these 12 unidirectional migrations, the fish was detected on all four receivers once, on three receivers eight times, on two receivers twice and on one receiver once. Variation in detections followed no apparent pattern, and detections were identical for two consecutive migrations only once. The number of detections on any one receiver during a migration was low (3.4±1.8 detections; *mean*±*s*.*e*.) further highlighting the likelihood of passing receivers undetected. Migration routes presented here represent the most complete detected migration for each individual (figure 1).

**Figure 3.**
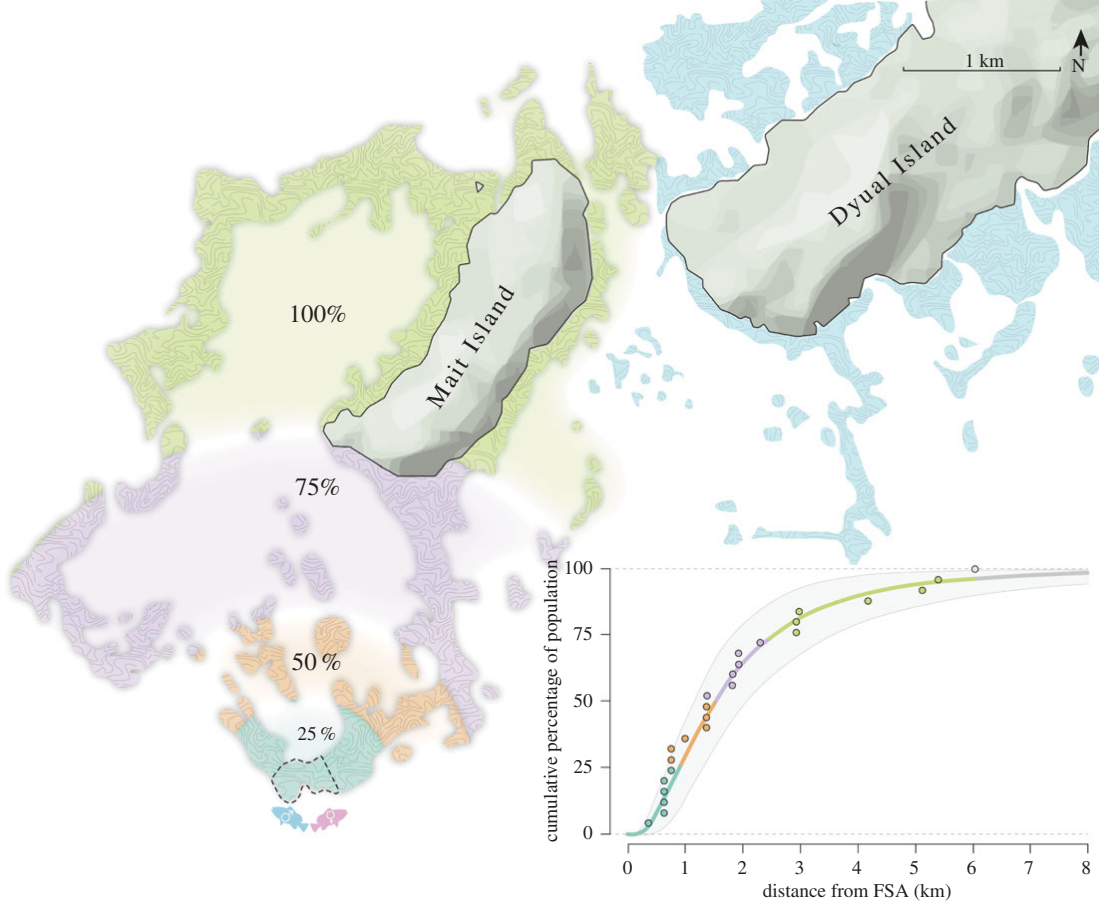
Area of catchment quantiles for *E. fuscoguttatus* from the Bolsurik FSA site, for 25 (aqua blue), 50 (orange) and 75 (purple) per cent of the spawning population, as calculated by the migration kernel (inset), and minimum complete catchment (green) calculated using the minimum convex polygon fitted over all locations of acoustic detection or recapture. Dashed line delineates current LMMA area. Inset: cumulative migration kernel. Points represent maximum recorded Euclidean migration distance for each individual, from either acoustic detection or recapture by the local fishery. The maximum-likelihood fit of the lognormal function to the uncorrected dataset (*μ*=0.40, *σ*=0.74) is plotted, coloured to match mapped catchment quantiles. Solid light-grey area represents 95% bootstrapped confidence band.

**Figure 4.**
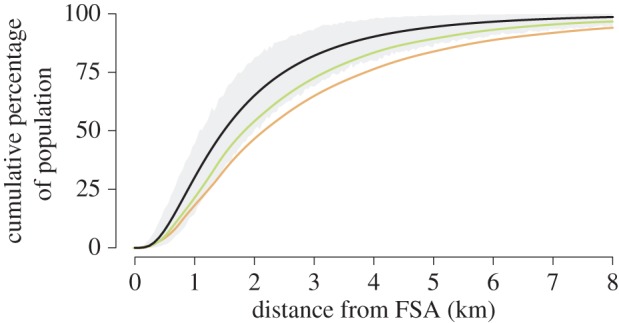
Comparison of maximum-likelihood migration kernels (each *n*=25). The uncorrected dataset maximum-likelihood fit of the lognormal function (*μ*=0.40, *σ*=0.76) is plotted (black line), with 95% bootstrapped confidence interval (grey area). The maximum-likelihood fit of the lognormal functions are also plotted for the ‘1.5 corrected’ (*μ*=0.63, *σ*=0.79; green line) and ‘2.0 corrected’ (*μ*=0.79, *σ*=0.84; orange line) datasets.

Maximum recorded migration distances reached 6 km but were skewed towards shorter distances (table 1 and figure 3). There was no correlation either between migration distance and number of visits to the FSA (*r*=0.196, *n*=24, *p*=0.348), or between migration distance and total residence time at the FSA (*r*=0.285, *n*=24, *p*=0.168). The maximum-likelihood fit of the lognormal function fitted to the uncorrected dataset (*μ*=0.40, *σ*=0.76), suggested that 50% of *E. fuscoguttatus* that aggregate to spawn at the Bolsurik FSA remain within 1.5 km of the FSA site throughout the non-spawning season (within an area of 2.1 km^2^; table 1). The functions fitted to the dataset following application of correction factors of 1.5 (*μ*=0.63, *σ*=0.79) and 2.0 (*μ*=0.79, *σ*=0.84), reduce this to 39% and 32%, respectively (figure 4).

**Table 1.** Area required for the protection of percentages of the spawning population of *Epinephelus fuscoguttatus* at Dyual Island, Papua New Guinea. (For the complete (100%) population estimates; maximum migration distance is the maximum recorded distance from the FSA to the site of recapture of a tagged fish, and required MPA area is calculated by fitting a minimum convex polygon over all detections and recaptures. For all other (25–75%) population estimates—maximum migration distances represent the greatest Euclidean distance from the FSA as predicted by the lognormal migration kernel fit to the uncorrected dataset (maximum-likelihood fit), required MPA area represents the intersect of a circle of this radius centred on the FSA, and the complete population catchment (presented in square kilometres and as a percentage of the complete catchment area).)

	max. migration (km)		required MPA area (km^2^)		catchment protected (%)	
population (%)	max. likelihood	range (95% confidence)	max. likelihood	range (95% confidence)	max. likelihood	range (95% confidence)
25	0.90	0.62–1.24	0.78	0.69–1.81	5	4–11
50	1.49	1.13–1.97	2.12	1.52–4.30	13	9–27
75	2.45	1.77–3.34	8.21	3.44–9.61	51	21–59
100	6.03	n.a	16.19	n.a	100	n.a

### 3.3 Detections during non-spawning periods

Excluding detections from migratory movements (within 48 h of detection at the FSA), eight individuals (six males and two females) were detected during the non-spawning season. No individual was detected on multiple receivers, and detection frequency varied substantially between individuals, but with one exception was relatively consistent within individuals, suggesting high site fidelity during non-spawning periods. For example, three males were detected on one receiver throughout the non-spawning period of 2013 (September–December inclusive). The first individual was detected 22 524 times, with a mean time between detections of 14.3 min (±0.4) and only one undetected period greater than 1 day (1.3 days). The second individual was detected 49 times, with 23 undetected periods greater than a day (5.1±0.75 days; mean ± s.e.). The third individual was detected seven times, a mean 18.6 (±8.5) days apart. Only one individual showed substantial variability in detection over time; female F0056 was detected at receiver E1 for 9 days post-tagging, disappeared from the array for 84 days, returned to E1 for 69 days, before disappearing permanently.

### 3.4 Stakeholder surveys

Although less than half (45%) of respondents reported initial support for the Bolsurik LMMA in 2004, most stated that it had subsequently been beneficial to their livelihoods (80%), the community (93%) and the environment (97%), and all respondents expressed their current support for the LMMA in 2014. The majority of respondents (72%) suggested that current fisheries management practices could be improved (table 2). Implementation or improvement of enforcement infrastructure was the most commonly reported management issue (38%). Half of respondents suggested further spatial management, either through expansion of the no-take LMMA area (34%) or the creation of new periodically harvested LMMAs (19%). Sixteen per cent of respondents suggested opening the current no-take LMMA periodically for harvest by local fishers. Although a quarter of respondents (26%) believed that a system existed to punish violators, no respondent could recall a punishment being administered. Key informants confirmed that no enforcement system existed. A majority of respondents (59%) reported that poaching had occurred within the LMMA within the past 3 years, 18% reported personally witnessing poaching and 9% reported participating in poaching within the LMMA. Respondents reported that all poaching was carried out by members of the communities of western Dyual, who were known to them.

**Table 2.** Potential changes to current fisheries management at Dyual Island, Papua New Guinea, categorized and ordered by percentage of survey respondents (*n*=32) suggesting the change.

category	action	respondents (%)
institutional	strengthen enforcement	37.5
	increase awareness of management	15.6
	form management committee	9.4
spatial/temporal	expand current no-take LMMA	34.4
	create new periodically harvested closures	18.8
	open current no-take LMMA for periodic harvest	15.6
other	introduce size limits	12.5
	promote alternative livelihoods	9.4
	introduce effort restrictions	6.3
	introduce gear restrictions	3.1
no change		28.1

## 4. Discussion

Based on movement patterns evidenced by a combination of acoustic telemetry and tag-return data, the spatial ecology of *E. fuscoguttatus* aggregating to spawn at Bolsurik strongly supports their community-based management. The current LMMA is sized to effectively protect the spawning aggregation site. Additionally, although protection of the entire FSA catchment area is socially unrealistic, a relatively modest expansion of the current spatial protection would protect a substantial proportion of the spawning population through the non-spawning season. Surveys of local stakeholders suggest that such an expansion would be well supported.

Our study showed that the current LMMA was highly effective in protecting the spawning stock during spawning events; with two possible exceptions, all tagged *E. fuscoguttatus* that aggregated to spawn at the FSA remained within the LMMA boundaries throughout their visits. However, individuals were protected within the no-take LMMA for a relatively small proportion of the time; males and females spent around 95% and 98% of the 2 year study period within the openly fished area, respectively. Notably, one male returned to the FSA during 11 spawning months, yet still resided outside of the LMMA for 88% of the study period. The importance of protecting FSA sites is well established, and cannot be overstated [16]; however, this study further emphasizes the importance of management encompassing, but not limited to, the FSA site.

Maximum recorded migration distances demonstrate that the catchment area of the FSA (i.e. the total area used by the spawning population throughout the year) is at least 16 km^2^. This is considerably smaller than the only other catchment area calculated for this species, in Pohnpei (100–175 km^2^), suggesting substantial geographical variability in migration distances [19]. Nonetheless, a spatial closure of 16 km^2^ is almost certainly socially untenable here or elsewhere where fishers with low spatial mobility are highly dependent on coral reef fisheries. The migration kernel illustrates however, that modest spatial expansion of the LMMA could offer relatively large conservation gains. For example, expansion of the current 0.2 km^2^ LMMA to the Pacific median size of 1 km^2^ would protect approximately 30% of the population during non-spawning periods, and increasing the LMMA to 2 km^2^ would protect approximately half of the *E. fuscoguttatus* population throughout the non-spawning season. This is 4.8- and 3.8-fold greater than expected if the population was evenly spread throughout the catchment area. Importantly, the conservation return on expanding spatial protection diminishes markedly as the full catchment area is approached, such that the area required to protect the quarter of the population furthest from the FSA is 10-fold greater than that required to protect the closest quarter. Although further studies are needed before extrapolating to additional FSAs and species, this result demonstrates that a more detailed understanding of migration patterns is required before rejecting the use of spatially restricted management tools.

It must be noted that all migration data reported here represent minimum distances. This is problematic if the migration distances are significantly underestimated. Underestimation of migration distances may occur through at least two, non-mutually exclusive mechanisms. First, if the number and length of visitations to the FSA were inversely correlated with migration distance (i.e. individuals that reside closer to the FSA spend more time at the FSA), then individuals that migrate shorter distances would probably be over-represented in the tagged sample. However, no such correlation was observed either here, or in a previous study (K. L. Rhodes 2016, personal communication). Second, migration distance data may be ‘distance weighted’ owing to sampling bias [38]. Specifically, likelihood of acoustic detection was presumably higher proximal to the FSA—owing to increased receiver coverage. Ideally, distance weighting could be reduced through design (e.g. with acoustic receivers deployed in a grid formation). However, greater coverage was required proximal to the FSA to accurately determine visitation patterns and to delineate migration corridors. Additionally, receiver placement was considerably constrained by the complex bathymetry and patchy habitat at the site. Thus, limited resources, logistic constraints and balancing multiple objectives outlined prior to the study limited the feasibility of eliminating distance weighting by design.

Where distance weighting cannot be eliminated through study design, correction factors can be used to assess its effect on observed movement patterns [38]. However, relatively consistent acoustic detection patterns and the absence of detections across multiple receivers during the non-spawning season, both here, and in a previous study (K. L. Rhodes 2016, unpublished data), suggest high non-spawning site fidelity for *E. fuscoguttatus*. Thus, data from detections or recaptures during the non-spawning season (i.e. excluding individuals with data from migratory movements only) (44%) are likely to represent accurate migration distances. Correction factors of 1.5 and 2.0 were therefore applied to the data from migratory periods only (56%). Uniform correction factors were applied, because more complex distance-dependent correction factors are based on numerous assumptions which are unlikely to be met in this complex system [38]. Even after applying a correction factor of 2.0 (effectively doubling detected migratory movement distances), the migration kernel suggested that expansion of the LMMA to 2 km^2^ would protect 32% of spawning individuals (figure 4). Further, as 32% of all tagged individuals were detected or captured within this area during the non-spawning season, we suggest that this represents a conservative minimum estimate.

Local stakeholder support for the fishing closure at Bolsurik increased substantially over the decade following LMMA implementation, and all survey respondents reported their current support for the LMMA. Additionally, there was strong support for expanding spatial protection of the current Bolsurik LMMA, with 34% of respondents suggesting this change. However, 16% of respondents suggested that the LMMA be opened periodically for harvest by community members. There is certainly ample opportunity to seasonally harvest the FSA; acoustic data demonstrate that *E. fuscoguttatus* aggregate here during at least seven lunar months annually, substantially more than the three to four lunar months recorded elsewhere for this species [19,39,40]. However, we do not endorse periodic harvest of this FSA for the following reasons. First, because of the high rates of return by individual fish to the FSA—particularly males—a substantial proportion of the total spawning stock could be removed in a single month. Second, currently no effective mechanism exists for enforcing community regulations, as emphasized by local stakeholders. For this reason, it would probably prove difficult to reinstate the closure post-harvest [13]. Finally, our limited understanding of recruitment dynamics, temporally variable impacts from fishing, and reproductive output relative to seasonal spawning times, make fine-scale harvest recommendations unjustified at this time. Additional research that improves on our current understanding of these factors may in the future allow recommendations on limited FSA fishing to occur.

Instituting a system of graduated sanctions on violators (increasing in severity with each successive infringement) is one of the key design principles for sustained community-based management success [3,41], and should thus be prioritized here. Household surveys suggest strong local support for such action. We further advocate that the local community continues to adopt a precautionary approach towards managing their grouper fisheries by continuing the permanent closure of the FSA site while also expanding the LMMA to partially protect the stock throughout the non-spawning period. The migration kernel demonstrates that the current LMMA provides negligible protection during the non-spawning season. While the migration kernel allows for the calculation of the optimal population protection by area, further investigation of population recovery rates and fishery pressure is required to assess the socially and ecologically appropriate LMMA size here. However, in the absence of such data, the expansion of the LMMA to encompass 1–2 km^2^ would protect 30–50% of the spawning population year-round, with the additional benefit of protecting common migration corridors (figure 1)—a key management priority for this species [24]. A spatial expansion of this magnitude is also unlikely to increase the burden of enforcement here, as current and proposed LMMA areas are equally visible from the surrounding area. Any locally administered system of sanctions is more likely to involve reports by witnesses to infringement, than the apprehension of alleged poachers in the act. According to household surveys, all poaching at the site within the past 3 years was carried out by members of the local community, and identification of poachers was not difficult.

It should be noted that these recommendations are neither intended to be exhaustive, nor to imply their superiority in achieving social or ecological goals. The establishment of a system of enforcement and the spatial expansion of the current LMMA must be considered among a host of management measures, such as seasonal closures, and restrictions on gear and effort—some of which have some existing stakeholder support (table 2).

This study provides promising evidence that species which are known to migrate moderate distances may still be conserved using community-based spatial management. The diminishing returns as spatial protection approached the entirety of the spawning population highlights the importance of understanding patterns of migration, over simple population boundaries. While direct comparisons with socially acceptable scales of management are still missing, the area required to protect 30–50% of the spawning population here is relatively congruous with community-based LMMAs throughout Papua New Guinea, and the wider region. This study thus provides initial evidence that designing LMMAs centred over, but not limited to, FSAs can provide robust conservation benefits, relative to area protected.

## Data accessibility

All data and scripts necessary to fully reproduce the analyses described in this paper are freely available at http://dx.doi.org/10.5061/dryad.26j85.
